# Mapping the olfactory mindscape in museums: a grounded theory investigation into smellscape perception and its impact on cultural tourism experience

**DOI:** 10.3389/fpsyg.2026.1776370

**Published:** 2026-05-26

**Authors:** Xi Ling, Menlixue Cheng

**Affiliations:** Faculty of Architecture and City Planning, Kunming University of Science and Technology, Kunming, Yunnan, China

**Keywords:** smellscape, olfactory perception, grounded theory, sensory museology, cultural tourism, multisensory experience, visitor emotion

## Abstract

Ethnographic museums are increasingly visited as cultural tourism destinations, yet their exhibition design remains predominantly visual, overlooking the potential of smell to shape tourists’ emotional engagement, memory, and well-being. While smellscape research has grown in environmental psychology, a process-oriented model explaining how visitors perceive and interpret culturally-coded odors in museum settings is still lacking. This study aims to investigate the cognitive-affective process underlying tourists’ smellscape perception in an ethnographic museum and to construct a theoretical model that explains how olfactory experiences influence cultural meaning-making and behavioral responses. Adopting a constructivist grounded theory approach, we integrated participatory smell-walks with in-depth semi-structured interviews. Data were collected from 120 visitors at the Yunnan Ethnographic Museum (China). All participants completed a smell-walk and survey; 30 participated in follow-up interviews. Analysis followed open, axial, and selective coding using NVivo, with member-checking and researcher triangulation ensuring rigor. The analysis yielded a five-category theoretical model: (1) Smell Perception—detection and description of odors; (2) Smell Association—cognitive links to behaviors, memories, and environments (behavioral, subjective, perceptual, environmental); (3) Smellscape Evaluation—assessments of intensity, appropriateness, preference, safety, and harmony; (4) Environmental Expectation—desires for authenticity, atmospheric resonance, and emotional engagement; and (5) Cultural Export—potential for heritage innovation and enhanced tourist experience. The model delineates a dynamic pathway from sensory input to meaning-making and behavioral outcomes. This study provides the first grounded theory model of smellscape perception in an ethnographic museum from a tourism perspective. It advances sensory tourism research by demonstrating how strategically curated smellscapes can deepen emotional engagement, strengthen cultural memory, and enhance visitor well-being. The findings offer an evidence-based framework for designing multisensory heritage experiences that move beyond visual-centric practices, with direct implications for museum management and destination marketing.

## 1 Introduction

Ethnographic museums serve as pivotal custodians of tangible and intangible heritage (Chang, 2023; ICOM 2021). In the context of cultural tourism, these institutions are increasingly positioned as destination experiences where visitors seek not only knowledge but also emotional resonance and memorable encounters (Pine and Gilmore, 1999). However, prevailing curatorial paradigms remain overwhelmingly ocularcentric, prioritizing visual artifacts while marginalizing other sensory modalities (Classen et al., 1994; Howes, 2021; Wartmann et al., 2021). This sensory imbalance overlooks the profound role of olfaction in human cognition, memory, and emotional engagement—a disconnect that undermines holistic cultural immersion (Herz, 2004; Stevenson, 2014; Axel, 1995; Willander and Larsson, 2007).

The concept of “smellscape”—the olfactory dimension of a place as perceived by people—provides a critical framework (Porteous, 1985; Henshaw, 2013; Wu, 2022; Diaconu et al., 2011; Chen and Yuan, 2017). As Xiao et al. (2021) document, smellscape research has grown exponentially over the past decade, with three main themes: (a) intangible heritage and historical reconstructions, (b) therapeutic and experiential elements (including tourism and well-being), and (c) design approaches for the built environment. Museums are explicitly identified as key contexts for applying smellscape methodologies (Xiao et al., 2021).

Within museum studies, pioneering work has recognized scent’s value in enhancing historical context, visitor engagement, and inclusivity, particularly for visually impaired audiences (Chen et al., 2024; Verbeek, 2016; Spence, 2020a). Levent and Pascual-Leone (2014) in the Multisensory Museum argue that engaging multiple senses transforms passive viewing into active, embodied experience. Pallasmaa (2005) emphasizes that architecture and exhibition spaces are fundamentally sensed through the whole body, with smell playing a crucial but often ignored role in place-attachment and memory. Despite this progress, research within ethnographic museums specifically remains nascent.

This context presents a distinct gap. In ethnographic museums, smells are not merely ambient but are semantically linked to cultural materials—the earthiness of pottery, the woodiness of textiles, the vegetal notes of ritual ingredients. These odors act as non-verbal signifiers of craft processes and symbolic knowledge systems (Kühne et al., 2021). For tourists, such olfactory cues can trigger powerful emotional responses, enhance destination image, and create lasting autobiographical memories (Xiao et al., 2021). According to the UNWTO (World Tourism Organization) (2019), cultural tourism accounts for nearly 40% of global tourism, and ethnographic museums are among the most visited attractions in many regions. Yet, no coherent theoretical model explains how tourists process these culturally-coded smells.

Current research lacks a model capturing the dynamic psychological process through which visitors perceive, interpret, evaluate, and derive meaning from olfactory cues. To address this, our study poses the central research question: What is the process by which tourists perceive, cognitively process, and derive meaning from the smellscape within an ethnographic museum, and how does this process shape emotional and behavioral responses?

Employing constructivist grounded theory (Charmaz, 2006; Corbin and Strauss, 2014), we generate an emergent model from data collected at the Yunnan Ethnographic Museum. This approach allows a bottom-up, visitor-centered understanding. The model aims to contribute to environmental psychology, sensory museology, and tourism studies by delineating the cognitive architecture of smellscape perception and offering an evidence-based foundation for designing multisensory heritage experiences.

## 2 Materials and methods

### 2.1 Research design and philosophical stance

This study adopted an exploratory qualitative design underpinned by a constructivist paradigm, guided by the methodological principles of grounded theory (Charmaz, 2006; Corbin and Strauss, 2014; Glaser and Strauss, 1967). This approach is ideally suited for investigating complex, subjective phenomena like sensory perception, as it facilitates the generation of theory directly from data, acknowledging the co-construction of meaning between participant and researcher. The study protocol received ethical approval from the Institutional Review Board of Kunming University of Science and Technology (Ethics Number: KMUST-MEC-2025-155), and all procedures adhered to the principles of the Declaration of Helsinki. Written informed consent was obtained from all participants.

### 2.2 Study context

The research was conducted at the Yunnan Ethnographic Museum (Kunming, China), a national first-class museum and the largest of its kind in China (National Cultural Heritage Administration, 2020; Zhao et al., 2023). As a key destination for domestic and international tourists seeking authentic ethnic cultural experiences, the museum receives over 500,000 visitors annually. Its collection of over 40,000 artifacts from 25 ethnic groups, displayed across 11 thematic halls within a traditional courtyard-style complex, provides a rich olfactory environment.

### 2.3 Participants and recruitment

A purposive sampling strategy was employed to recruit 120 adult tourists/visitors, ensuring diversity across gender (60 female, 60 male), age (M = 34.2, SD = 8.7, range 20–60), ethnicity (90 Han, 30 from ethnic minorities), and origin (50 local residents, 70 non-local tourists). Purposive sampling was chosen to capture maximum variation in demographic characteristics relevant to cultural tourism (Patton, 2015). All 120 completed a smell-walk and survey. From this pool, a subset of 30 participants was selected for in-depth semi-structured interviews to achieve theoretical saturation (Guest et al., 2006), maintaining proportional demographic representation. The remaining 90 provided survey data used for demographic profiling and initial odor frequency analysis.

### 2.4 Data collection procedures

Data collection involved a multi-stage, integrated protocol designed to capture both broad patterns and deep subjective experiences.

**Figure 1:**
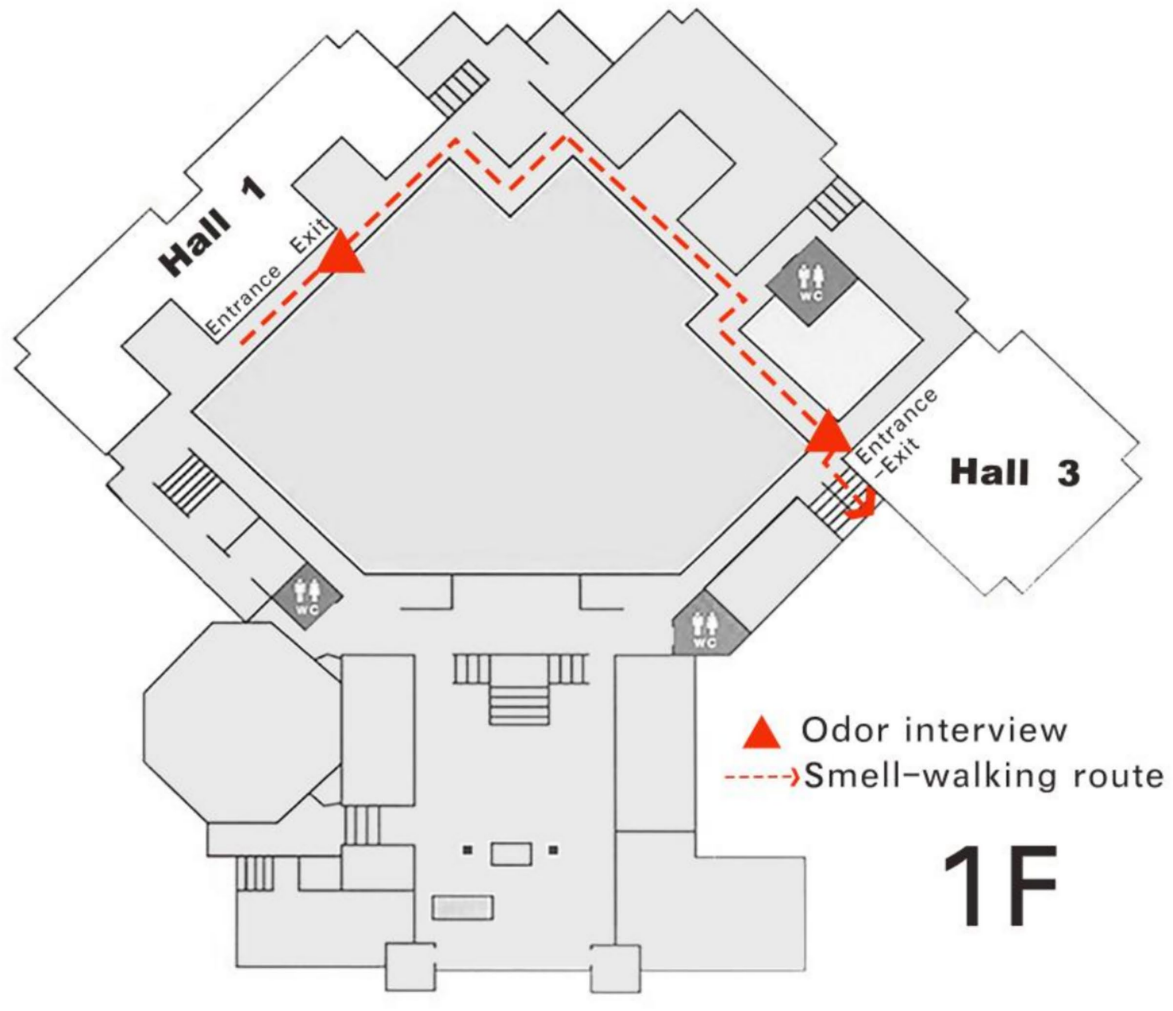
The smell walking route on the first floor of the Yunnan Ethnographic Museum.

**Figure 2:**
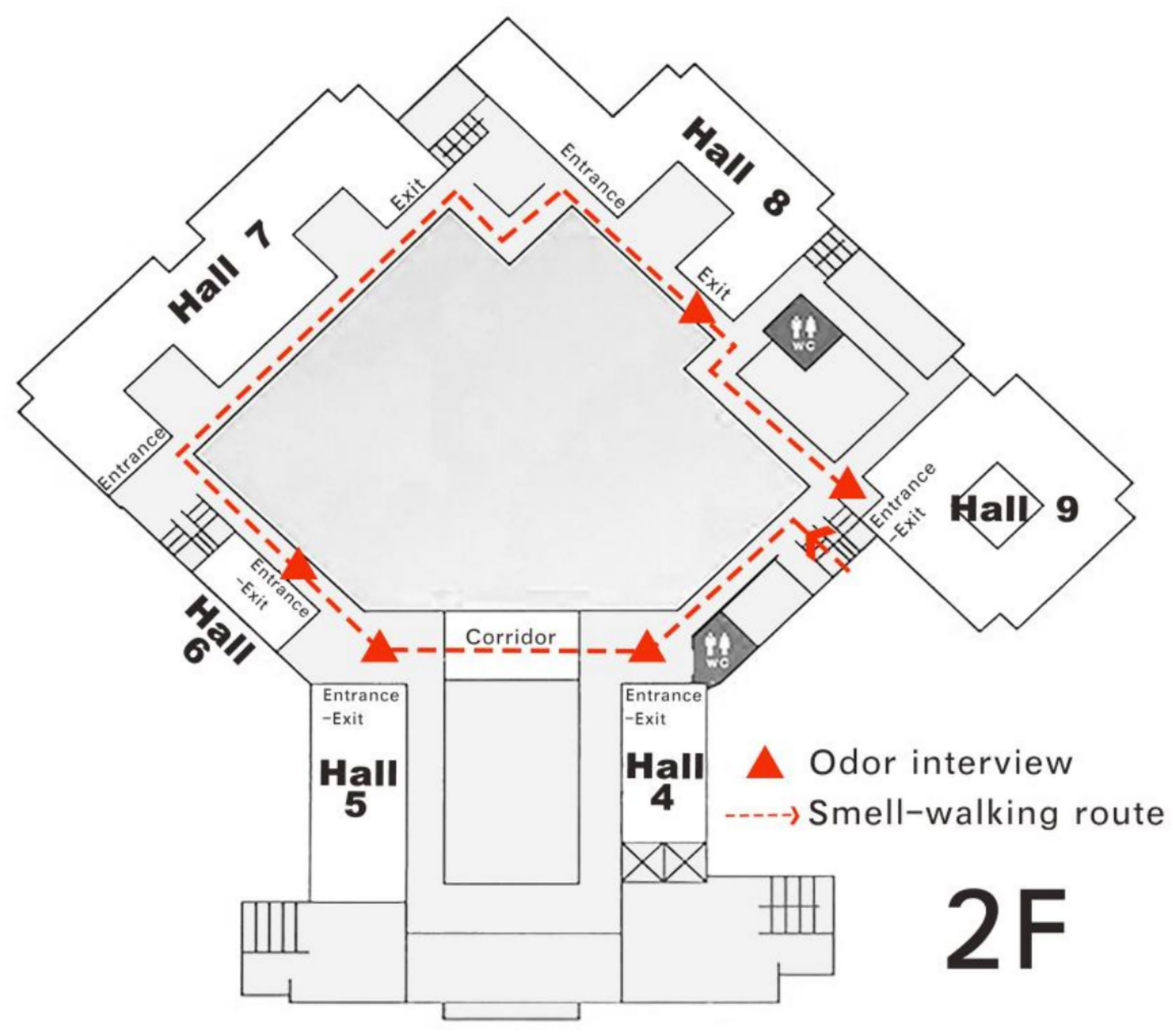
The smell walking route on the second floor of the Yunnan Ethnographic Museum.

*Preliminary scoping and route design:* Initial site visits mapped exhibition themes, visitor circulation, and potential odor zones, informing a standardized 1.3 km smell-walking route covering seven core halls (Figures 1, 2).

*Stage 1: smell-walk and survey:* Each participant was individually accompanied by a researcher along the predefined route. They were instructed to verbalize any odors detected spontaneously (Bouchard, 2013; Xi, et al., 2022). The researcher recorded detailed field notes on descriptors, location, intensity, and immediate associations using a standardized journal. Immediately following the walk, participants *completed a brief survey capturing demographic data and initial impressions*.

**Figure 3:**
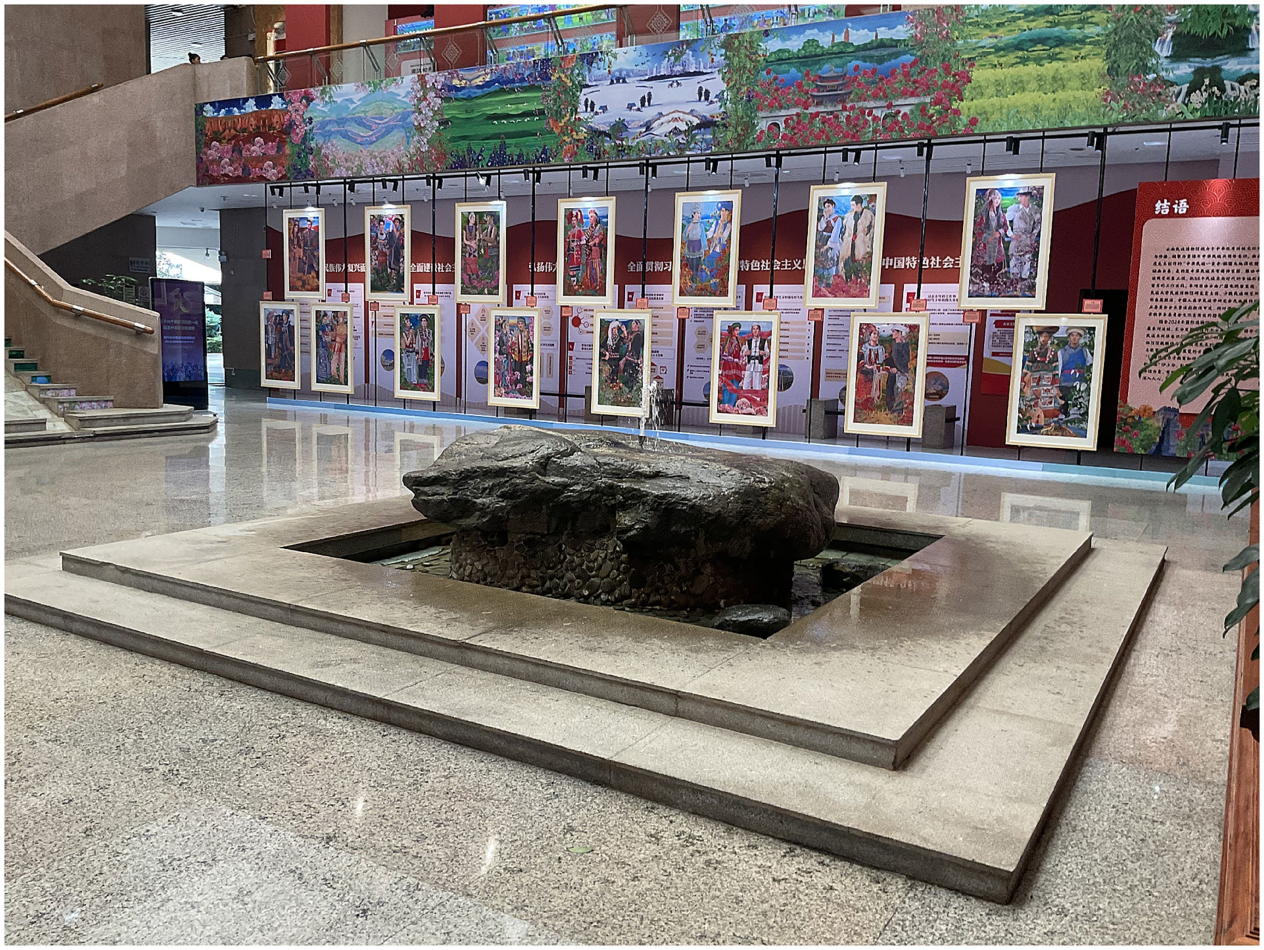
Photographs of smell zones.

*Stage 1: smell-walk and survey*: Each participant was accompanied by a researcher and instructed to verbalize any odors detected spontaneously. The researcher recorded field notes on descriptors, location, intensity, and associations. Photographs of smell zones were taken (Figure 3). Immediately following, participants completed a brief survey capturing demographics and initial impressions.

*Stage 2: semi-structured interviews:* A subset of 30 participants engaged in audio-recorded interviews (mean 45 min). The interview guide (Table 1) was developed based on existing smellwalk protocols (Henshaw, 2013; McLean, 2016a,b) and pilot testing, covering four domains: (a) descriptive recall; (b) cognitive/affective responses; (c) evaluative judgments; (d) reflective suggestions.

**Table 1:** Interview outline.

Interview topic	Purpose of problem	Related problem
Personal information	Understand basic information	Gender, ethnicity, local or tourist?
Smell concept and national cognition	Establish smell concepts and associations	What smell comes to mind first? What word would you use to describe the smell?
		What do you think is the smell of the ethnical? What factors will affect the change?
	Guide national smell cognition	What can you think of about the smell of the Dai nationality? Food, plants, folk culture, religious festivals, commemorative gifts, etc.
Smell perception in the museum	Recall and description of guided smell perception	Did you smell something when you visited this exhibition hall?
		How do you feel when you smell it? Can you think of any ethnical customs or things?
		What do you think are the causes of the odors in the museum that make you feel comfortable or uncomfortable?
		Have you ever seen or learned about an exhibit or national culture that led you to associate a certain smell with it?
Tap into the smell perception experience	Open discussion, in-depth understanding of the interviewee experience	Talk about your impression of Yunnan Ethnographic Museum as a whole.
		What do you think should be done to present ethnical smells in Ethnographic Museum?
		What are your suggestions and ideas for the future update and development of the Ethnographic Museum?

### 2.5 Data analysis

Data analysis followed grounded theory stages (Braun and Clarke, 2006; Corbin and Strauss, 2014) using NVivo 12. To enhance transparency, the first author conducted initial coding, and a second researcher independently coded 20% of transcripts, achieving 86% agreement; disagreements were resolved through discussion. (Bylund and Denzin, 1972; Che et al., 2023; Duan et al., 2020)

*Open coding*: interview transcripts and field notes were analyzed line-by-line to identify initial codes (e.g., “smells like old books,” “reminds me of a woodshop,” “too strong, felt stuffy”). Similar codes were constantly compared and grouped into conceptual categories (e.g., “Musty Odor,” “Behavioral Association,” “Negative Evaluation”).

*Axial coding*: relationships between these initial categories were examined and synthesized into higher-order, abstract main categories (e.g., “Smell Perception,” “Smell Association”). The properties (characteristics) and dimensions (variations) of each category were defined and elaborated.

*Selective coding*: the core category—“The Process of Smellscape Perception in an Ethnographic Museum”—was identified. All other main categories were systematically related to this core to construct a coherent explanatory theoretical model.

*Theoretical saturation and validation*: coding continued until no new properties emerged after 25 interviews; the remaining 5 confirmed saturation. Member-checking with two participants affirmed model resonance.

*Researcher reflexivity*: the authors, both trained in architecture and environmental psychology, approached the study with interest in multisensory design but bracketed assumptions by maintaining reflexive memos throughout.

## 3 Results

### 3.1 Participant demographics

The 120 participants comprised 60 males and 60 females. Age distribution: 30 (20–30), 80 (31–40), and 10 (41–60). Among them, 50 were local residents, 70 were tourists, 90 were Han, and 30 were from ethnic minorities (Table 2).

**Table 2:** Background analysis of participants.

Social characteristics	Classification	Number
Gender	Female	60
	Male	60
Age	20–30	30
	31–40	80
	41–60	10
Source	Native	50
	Tourist	70
Nationality	The Han nationality	90
	The Ethnic minorities^a^	30

a These are the ethnic minorities unique to China, and a large number of them live in Yunnan Province of China.

### 3.2 The identified museum smellscape

Through smell-walking, participants identified six odor types. Below are representative verbatim quotes (participant IDs and identities provided):

“As soon as I entered the textile hall, there was this earthy, almost dusty smell—like old fabric stored for years. It made me think of my grandmother’s attic.” (P23, female, tourist).

“In the pottery section, I could smell burnt clay. It was not strong, but very distinctive. I imagined the firing process.” (P45, male, local).

“The disinfectant smell in some corridors was too strong. It felt like a hospital, not a museum. That really broke the atmosphere.” (P67, female, tourist).

“There was a sweet, woody smell near the bamboo exhibits—very natural, like a forest.” (P12, male, tourist).

The six types are:

1. Earthy/Mineral: Dust, clay, stone (associated with pottery, tiles, building materials).
2. Musty/Old: Aged paper, textiles, closed spaces (associated with documents, stored costumes).
3. Woody: Fresh wood, veneer, bamboo (associated with architecture, tools, displays).
4. Metallic: Rust, metal polish (associated with jewelry, instruments).
5. Vegetal/Botanical: Plants from adjacent gardens, herbal notes (associated with environment, some artifacts).
6. Chemical/Operational: Disinfectant, insecticide, cleaning agents (associated with museum maintenance).

### 3.3 The smellscape perception model

**Figure 4:**
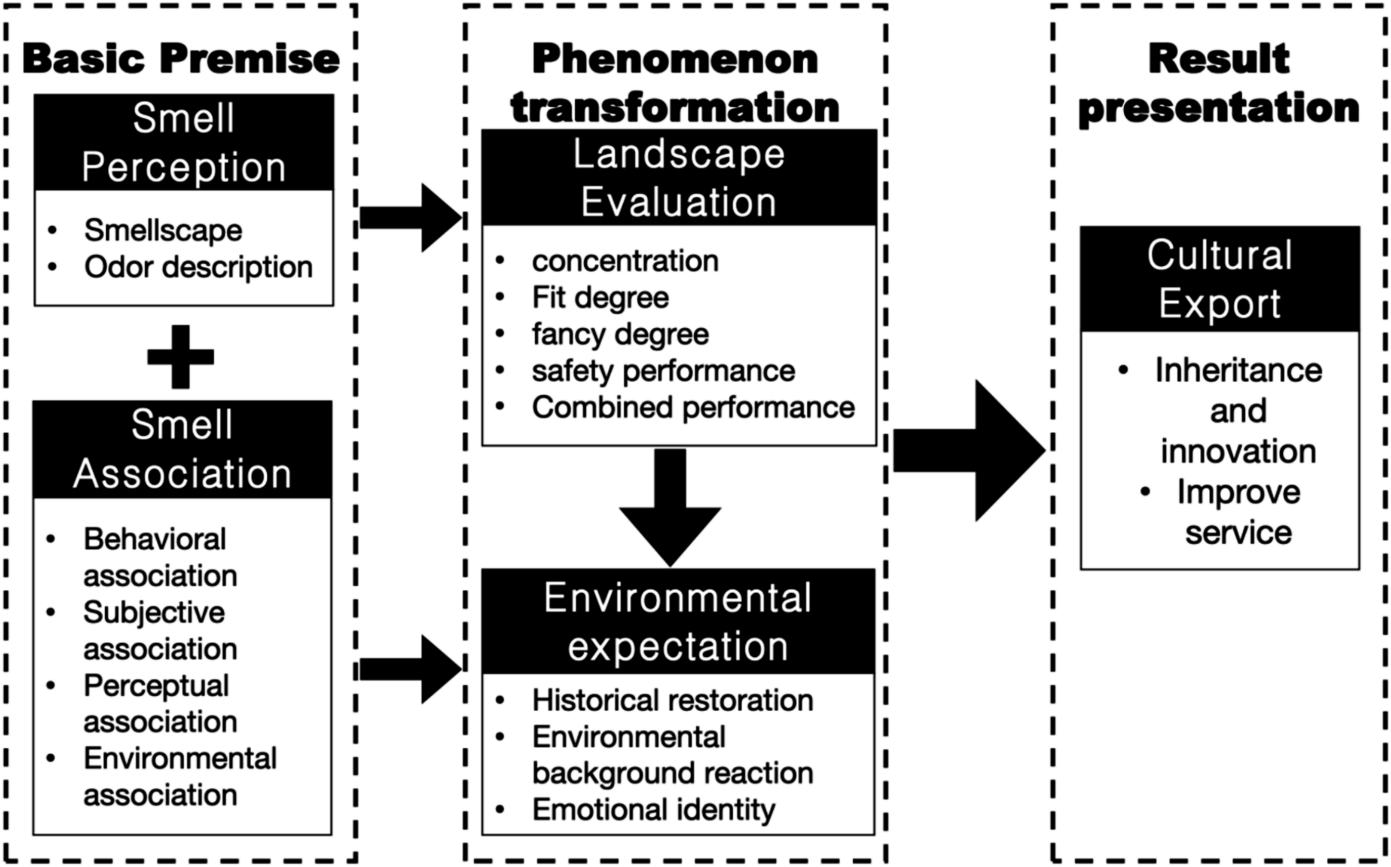
Perceptual model of the smellscape of the Ethnographic Museum.

Grounded theory analysis yielded a five-category model (Figure 4). Below we elaborate each category with supporting evidence.

#### 3.3.1 Smell perception

Foundational detection stage. Participants used descriptors like “pungent,” “faint,” “sweet.” This category emerged directly from open coding of sensory vocabulary.

#### 3.3.2 Smell association

Four subtypes emerged inductively:

Behavioral Association: “The smell of leather in the musical instrument hall made me think of someone stretching strings.” (P78, female, tourist).

Subjective Association: “Silver jewelry should smell clean and cold—that’s what I imagine.” (P34, male, local).

Perceptual Association: “Seeing the loom, I could almost smell the oil and thread friction.” (P56, female, tourist).

Environmental Association: “Those roof tiles—I smelled rain and wet soil, like a village after a storm.” (P89, male, tourist).

These associations align with Obrist et al.’s (2014) categories of everyday smell associations.

#### 3.3.3 Smellscape evaluation

**Figure 5:**
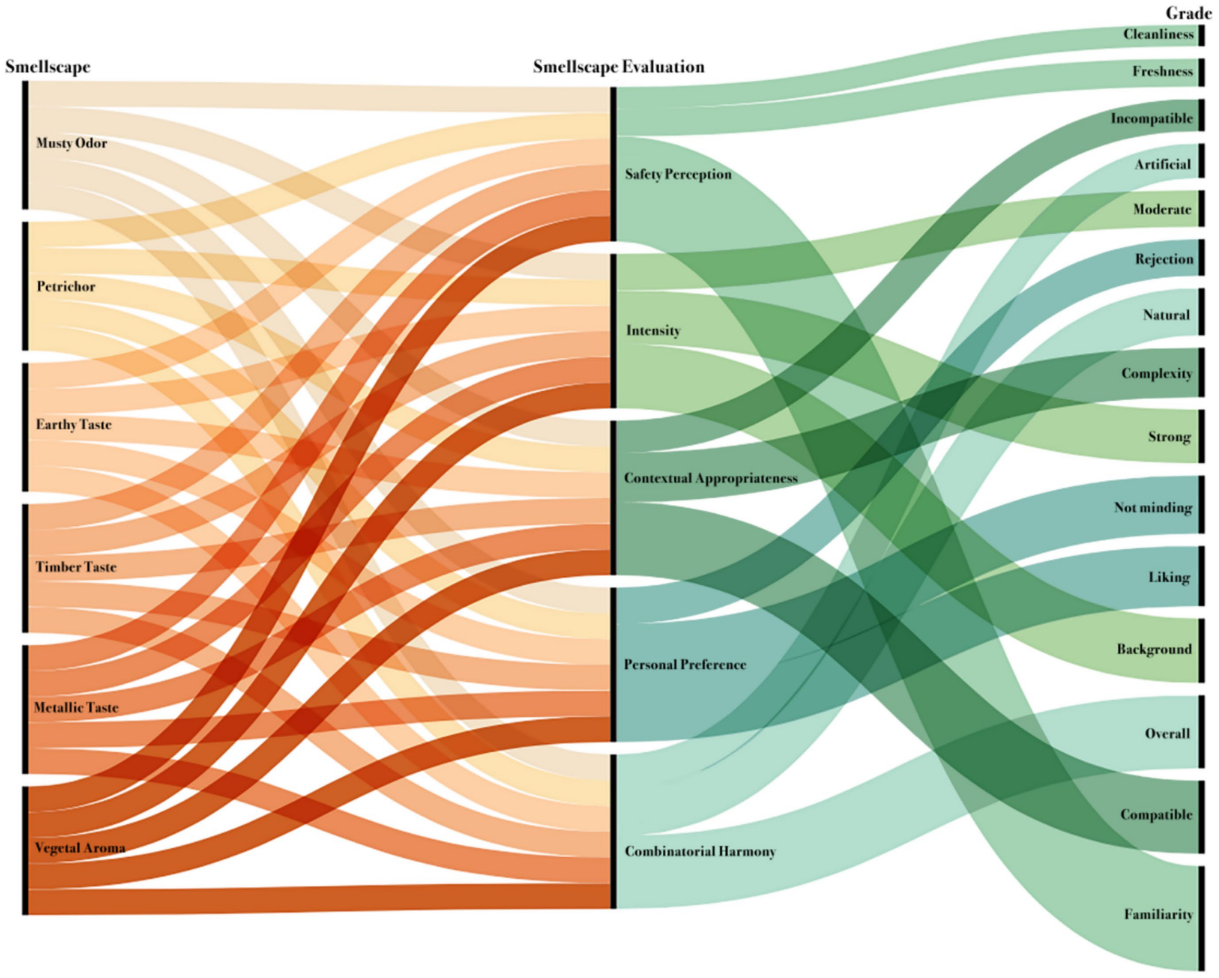
Grade proportion of smellscape evaluation.

Five dimensions rated by participants (Figure 5). Notably, 68% rated chemical odors as “incompatible,” and 72% found strong intensities “unpleasant.” Participants explained: “The disinfectant smell ruined the whole experience” (P67).

The subjective assessment of the olfactory experience along five dimensions:

Intensity: Perceived strength (Strong, Moderate, Background).

Contextual Appropriateness: Fit between odor and its setting (Compatible, Incompatible, Complex).

Personal Preference: Affective response (Liking, Neutral, Disliking).

Safety Perception: Feelings of familiarity, freshness, and cleanliness.

Combinatorial Harmony: Judgment of how multiple smells blend (Overall, Natural, Artificial harmony).

#### 3.3.4 Environmental expectation

This category emerged from participants’ explicit desires. For example: “I wish the museum smelled more like a real Dai village—bamboo, rice, maybe incense.” (P101, female, tourist); “The atmosphere feels dead. Smells could make it alive.” (P22, male, local). These led to three sub-dimensions: Historical Authenticity, Atmospheric Resonance, Emotional Engagement.

#### 3.3.5 Cultural export

Participants suggested olfactory storytelling: “If they released the scent of herbs used in rituals, I would remember that culture much better.” (P76, male, tourist). This became the final outcome category.

The model posits a dynamic relationship: Perception → Association → Evaluation → Expectation → Export.

## 4 Discussion

This study moves beyond merely identifying smells in a museum to modeling the complex perceptual and cognitive journey they provoke within a cultural tourism context. Our findings offer a grounded theory model that explains how tourists actively construct meaning from olfactory cues, and we discuss below the theoretical, methodological, and practical implications of this model.

### 4.1 Theoretical contributions

Our model advances smellscape theory by specifying its mechanisms within a culturally-charged, tourism-oriented space. While foundational work by Porteous (1985) and Xiao et al. (2018) described what a smellscape is, our model details how it is processed—from detection to association to evaluation to expectation to cultural outcome. This process-oriented perspective is novel for sensory museology (Classen et al., 1994; Levent and Pascual-Leone, 2014), which has rarely provided granular, stage-based models of sensory processing.

The Smell Association category is particularly significant. Multiple participants described how odors triggered rich mental narratives. For instance, one tourist explained: “The musty paper smell did not just smell old—it told me that these documents were touched by real people, long ago” (P33, female, tourist). Another participant noted: “When I smelled the leather in the instrument hall, I immediately pictured a craftsman working with his hands—it made the exhibit feel alive” (P78, female, tourist). A third added: “The earthy smell from the pottery did not come from any actual clay—but seeing the pots, I could almost smell the rain and soil” (P89, male, tourist). These accounts align with Obrist et al. (2014), who found that everyday smell associations are often narrative and context-dependent, and with Kühne et al. (2021) on the multisensory nature of cultural landscapes. Importantly, they demonstrate that smell associations are not idiosyncratic but follow predictable patterns (behavioral, subjective, perceptual, environmental) that can inform design.

The Evaluation dimension directly relates to tourist satisfaction and well-being. Participants who rated smells as “compatible” with the exhibition context reported higher enjoyment and willingness to recommend the museum. One visitor stated: “The woody scent near the bamboo displays felt perfectly natural—it made me want to stay longer and explore more” (P12, male, tourist). Conversely, strong chemical odors caused discomfort and even avoidance behavior. As one participant remarked: “The disinfectant smell in the corridor was so strong that I hurried through that section—it really broke the immersion” (P67, female, tourist). Another expressed: “I almost left because of the chemical smell; it felt like a hospital, not a museum” (P23, female, tourist). These findings echo Spence (2020) on ambient scent and well-being in built environments, and Xiao et al. (2021) on the need to consider both positive and negative odorants in smellscape design.

The Environmental Expectation category reveals that tourists are not passive recipients but actively desire authentic, engaging olfactory environments. This aligns with co-creation theory in tourism (Pine and Gilmore, 1999). One participant articulated this desire clearly: “I want to smell the past, not just see it. A museum should engage all my senses, otherwise it feels flat” (P12, male, tourist). Another shared: “The museum feels too sterile. If it had smells of cooking or incense, I would feel like I’m actually in a Dai village” (P101, female, tourist). A third participant added: “I came here to experience Yunnan’s culture—smell is a huge part of that. Without it, something is missing” (P56, female, tourist). These statements highlight a gap between current visual-centric exhibitions and tourists’ multisensory expectations, a gap that our model helps to address.

### 4.2 Methodological reflection

The integration of smell-walking with immediate interviewing captured both ephemeral sensation and reflective interpretation, mitigating recall bias. The use of participant verbatim quotes and photographic documentation (Figure 3) provides evidentiary depth (Lygum and Xiao, 2025). Following Xiao et al. (2021), we emphasize that smellwalk methodologies require careful attention to group size and context; our one-on-one approach minimized social desirability bias. Additionally, the high inter-coder agreement (86%) and member-checking with participants enhanced the credibility of our findings.

### 4.3 Practical implications for sensory tourism design

Our model translates directly into actionable strategies for museum managers and tourism authorities. First, curating intentional smellscapes based on Association types can enhance narrative engagement. For example, introducing a subtle pine resin scent in a woodcraft exhibit or a light herbal aroma in a traditional medicine display would trigger behavioral and environmental associations, as participants like P78 and P89 implicitly suggested. Second, managing evaluative dimensions is critical: overpowering maintenance odors rated as “incompatible” (e.g., disinfectant) should be replaced with culturally-framed alternatives such as herbal insect repellents traditionally used by ethnic groups. Ensuring safety through cleanliness and ventilation remains paramount, as participants like P67 and P23 explicitly linked discomfort to chemical smells. Third, fulfilling environmental expectations requires using scents with verifiable cultural provenance. One participant offered a concrete vision: “Release the smell of buckwheat pancakes during the Yi exhibition—that would be wonderful and so memorable” (P88, male, tourist). Another suggested: “A rice or bamboo scent in the Dai section would make me feel like I’m actually there” (P101, female, tourist). Fourth, leveraging digital olfaction—synchronized scent diffusion in AR/VR experiences—can create controlled, repeatable narrative moments, enhancing immersion and memorability (Spence, 2020a; Wuelser et al., 2022. Zhang et al., 2021; Zhou and Lin, 2022). Finally, creating olfactory pathways—themed routes such as “Smell of Craftsmanship” connecting pottery, textile, and instrument halls with complementary scents—would enrich the tourist journey, encourage longer stays, and deepen cultural learning (Tao and Duan, 2024).

### 4.4 Limitations and future research

This study has several limitations. The single-case design limits generalizability; findings from the Yunnan Ethnographic Museum may not fully apply to other museum types or cultural contexts. Participant sociocultural background (e.g., class, education, prior knowledge, olfactory sensitivity) was not systematically captured—a factor that may influence smell perception and should be addressed in future research. Additionally, while our sample included tourists and locals, we did not measure prior museum experience or travel motivation, which could moderate the observed relationships. Future research should: (1) quantitatively validate the model across multiple museums in different cultural settings; (2) experimentally test the impact of designed smellscapes on tourist memory, well-being, revisit intention, and willingness to pay; (3) explore cross-cultural and socioeconomic differences in smellscape perception; and (4) investigate how individual olfactory sensitivity (including post-COVID anosmia) moderates the perception–evaluation pathway.

## 5 Conclusion

This study set out to answer: What is the process by which tourists perceive, cognitively process, and derive meaning from the smellscape within an ethnographic museum? Through grounded theory analysis of smell-walks and interviews with 120 visitors at the Yunnan Ethnographic Museum, we constructed a five-category theoretical model: smell perception → smell association → smellscape evaluation → environmental expectation → cultural export.

Our findings demonstrate that olfactory experience in this cultural tourism context is not passive reception but an active chain of perception, associative interpretation, evaluative judgment, and expectation formation, ultimately feeding into the museum’s cultural mission and the tourist’s emotional and behavioral responses.

Theoretical contributions: (1) enriches smellscape theory with a process-oriented, context-specific framework; (2) bridges sensory museology and tourism studies by showing how olfactory cues shape destination image, authenticity perception, and memorability; (3) responds to Xiao et al.’s (2021) call for user-centered perceptual models in museum contexts.

Practical contributions: The model translates into concrete design principles—curation, evaluation management, expectation fulfillment, and technological integration—that can guide museums and tourism authorities toward intentional use of the olfactory dimension, thereby enhancing tourist satisfaction, well-being, and cultural learning.

As museums evolve from repositories to facilitators of immersive experiences, attention to the full sensorium is imperative. This research argues that the smellscape, particularly in culturally-rich settings like ethnographic museums, is a powerful yet delicate tool. When thoughtfully designed based on an understanding of visitor perception, it can profoundly deepen engagement, enhance memory, and give tourists a more authentic, holistic, and emotionally resonant connection to intangible heritage.

## Data Availability

The raw data supporting the conclusions of this article will be made available by the authors, without undue reservation.
